# Anti-inflammatory Properties of Stingless Bee Honey May Reduce the Severity of Pulmonary Manifestations in COVID-19 Infections?

**DOI:** 10.21315/mjms2020.27.3.16

**Published:** 2020-06-30

**Authors:** Ewe Seng Ch’ng, Thean Hock Tang

**Affiliations:** 1 Advanced Medical and Dental Institute, Universiti Sains Malaysia, Pulau Pinang, Malaysia; 2 School of Healthcare and Medical Sciences, Sunway University, Selangor, Malaysia; 3 Department of Biotechnology, Faculty of Applied Sciences, AIMST University, Kedah, Malaysia; 4 Public Health & Epidemiology Department, Nigerian Institute of Medical Research, Lagos, Nigeria

Dear Editor,

We read with great interests the speculation put forward by Mustafa MZ et al. (1) that stingless bee honey may act as a functional food to complement the treatment for Coronavirus Disease 2019 (COVID-19) caused by severe acute respiratory syndrome coronavirus 2 (SARS-CoV-2). Mustafa MZ et al. extrapolate the results of a few in vitro studies for the previously known coronaviruses including SARS coronavirus (SARS-CoV) to explain the interplay between bronchial epithelial cells and immune cells via various cytokines, especially IL-6 for the possible pathogenesis of SARS-CoV-2. This corroborates with the significantly elevated serum levels of IL-6 in non-survivor COVID-19 patients. Based on this model of disease progression focusing on overexpression of IL-6, stingless bee honey is proposed as a possible complementary treatment as honey has anti-inflammatory properties in tampering the IL-6 cascades.

Although much about pathogenesis of SARS-CoV-2 remains unknown, clinically staged progression of COVID-19 is being recognised and molecular mechanisms at each stage are being unveiled and inferred from other respiratory viral infections (2–5). In brief, in early infection stage I disease, inoculation and early establishment of disease occur with SARS-CoV-2 binding to epithelial cells in upper airway and conducting airway via angiotensin-converting enzyme 2 (ACE2) receptor. Local propagation of the virus with controlled innate immune response in this stage manifests clinically as mild and often non-specific symptoms such as fever and dry cough. Eighty-one percent of patients recovered from this stage (6). Symptomatic relief and antiviral treatment, if available, are main treatment rather than manipulating the host immune response.

In established pulmonary parenchyma stage II disease, infected epithelial cells undergo pyroptosis, a highly inflammatory form of programmed cell death, and release damage-associated and pathogen-associated molecular patterns, triggering a T helper 1 cell-polarised pro-inflammatory feedback loop involving neighbouring epithelial cells, endothelial cells, alveolar macrophages and T cells via production of pro-inflammatory cytokines and chemokines. Pulmonary damage and recruitment of immune cells manifests as viral pneumonia clinically with bilateral infiltrates or ground glass opacities on chest imaging. Hypoxia may occur in more advanced stage (stage IIb). At this stage, management primarily consists of hospitalisation with close observation, supportive care and antiviral therapy, if available. Only with hypoxia likely to require mechanical ventilation supports that anti-inflammatory therapy can be judiciously administered. About 14% of patients progressed to this stage (6).

Unfortunately, about 5% of patients developed stage III with systemic hyperinflammation syndrome (6). In this small population of patients, dysfunctional immune response triggers a cytokine storm with a myriad of cytokines such as tumour necrosis factor, IL-6, and IL-1β, causing not only widespread lung inflammation presenting as acute respiratory distress syndrome but also multiorgan failure. Treatment in this stage is tailored towards aggressive anti-inflammatory therapy to overcome the systemic inflammation. Clinical trials using corticosteroids (7) and IL-6 inhibitors such as tocilizumab (8) are underway.

In line with the staged progression of COVID-19, anti-inflammatory therapy might play roles in the more advanced stages of COVID-19 rather than in the early stages of the disease. As such, the ideas put forward by Mustafa MZ et al. to treat early infected patient with sting bee honey via its anti-inflammatory properties do not resonate well with the current understanding of COVID-19. Even in the later stages of COVID-19, it is doubtful sting bee honey has similar efficacy as compared to other anti-inflammatory therapies such as glucocorticoid administration.

Considering most of COVID-19 patients would manifest stage I disease and recover from this stage, it does not negate the preference of some patients for self-care treatments that may offer some benefits for symptomatic relief. These self-care treatments should be construed within the realms of symptomatic relief rather than specific treatments for COVID-19. In relation to this, guideline for acute cough associated with an upper respiratory tract infection or acute bronchitis, which usually has a viral etiology, states that honey as a self-care treatment may be used to alleviate cough symptoms (9), yet there is no strong evidence for or against the use of honey (10). Apart from honey, in fact, there is a long list of herbal therapies and dietary supplements that have empirical in vitro, in vivo or limited clinical trial evidence for prevention and treatment of respiratory viral infections (11). It would be clinically naïve to suggest every each of those herbal therapies and dietary supplements could potentially treat COVID-19 beyond the context of symptomatic relief.
